# Serodynamic Analysis of the Piglets Born from Sows Vaccinated with Modified Live Vaccine or E2 Subunit Vaccine for Classical Swine Fever

**DOI:** 10.3390/pathogens9060427

**Published:** 2020-05-29

**Authors:** Yi-Chia Li, Ming-Tang Chiou, Chao-Nan Lin

**Affiliations:** 1 Animal Disease Diagnostic Center, College of Veterinary Medicine, National Pingtung University of Science and Technology, Pingtung 91201, Taiwan; yichavet@gmail.com; 2 Department of Veterinary Medicine, College of Veterinary Medicine, National Pingtung University of Science and Technology, Pingtung 91201, Taiwan

**Keywords:** classical swine fever, porcine reproductive and respiratory syndrome virus, quantitative PCR, antibody, modified live vaccine, E2 subunit vaccine

## Abstract

Classical swine fever (CSF) caused by the CSF virus (CSFV) is one of the most important swine diseases, resulting in huge economic losses to the pig industry worldwide. Systematic vaccination is one of the most effective strategies for the prevention and control of this disease. Two main CSFV vaccines, the modified live vaccine (MLV) and the subunit E2 vaccine, are recommended. In Taiwan, CSF cases have not been reported since 2006, although systemic vaccination has been practiced for 70 years. Here, we examined the sero-dynamics of the piglets born from sows that received either the CSFV MLV or the E2 vaccine and investigated in the field the correlation between the porcine reproductive and respiratory syndrome virus (PRRSV) loads and levels of CSFV antibody. A total of 1398 serum samples from 42 PRRSV-positive farms were evaluated to determine the PRRSV loads by real-time PCR and to detect CSFV antibody levels by commercial ELISA. Upon comparing the two sow vaccination protocols (CSFV MLV vaccination at 4 weeks post-farrowing versus E2 vaccination at 4–5 weeks pre-farrowing), the lowest levels of CSFV antibody were found in piglets at 5–8 and 9–12 weeks of age for the MLV and E2 groups, respectively. Meanwhile, the appropriate time window for CSFV vaccination of offspring was at 5–8 and 9–12 weeks of age in the MLV and E2 groups, respectively. There was a very highly significant negative correlation between the PRRSV load and the level of CSFV antibody in the CSFV MLV vaccination group (*P* < 0.0001). The PRRSV detection rate in the pigs from the MLV group (27.78%) was significantly higher than that in pigs from the E2 group (21.32%) (*P* = 0.011). In addition, there was a significant difference (*P* = 0.019) in the PRRSV detection rate at 5–8 weeks of age between the MLV (42.15%) and E2 groups (29.79%). Our findings indicate that the vaccination of CSFV MLV in piglets during the PRRSV susceptibility period at 5–8 weeks of age may be overloading the piglet’s immune system and should be a critical concern for industrial pork production in the field.

## 1. Introduction

Classical swine fever (CSF) caused by the CSF virus (CSFV) is one of the most important swine diseases, resulting in huge economic losses to the pig industry worldwide, and it is a World Organization for Animal Health (OIE)-listed disease. CSFV (previously called hog cholera virus) belongs to the genus *Pestivirus* within the family *Flaviviridae* together with bovine viral diarrhea virus 1, bovine viral diarrhea virus 2 and border disease virus [1]. Recently, CSFV has been redesignated as *Pestivirus C* [1].

CSF is an immunosuppressive disease in which several immune escape mechanisms of CSFV have been reported, such as apoptosis, autophagy and pyroptosis in bone marrow hematopoietic cells, lymphocytes and lymphoid organs [2]. A low CD4/CD8 ratio has been observed in the peripheral blood mononuclear cells of infected fetuses and piglets challenged with either high- or low-virulence CSFV strains. A low CD4/CD8 ratio indicates dysregulation of the immune response [3]. During CSFV infection, the clinical signs mainly depend on the ages of pigs and the virulence of the viral strains. The clinical forms of CSFV can show acute, chronic and persistent courses. The persistent course usually requires infection of sows at approximately 50–70 days of pregnancy [4,5,6]. In general, the acute form of CSF leads to clinical and pathological features that are very similar to those of African swine fever [5,6]. In addition, CSF must also be considered in the differential diagnosis of erysipelas, porcine circovirus type 2 (PCV2)-associated diseases (PCVAD), salmonellosis and porcine reproductive and respiratory syndrome (PRRS) [6]. The overlapping of the clinical presentations may lead to a misdiagnosis of CSF as PRRS virus (PRRSV) infection. PRRSV infection also causes reproductive symptoms in gestational sows and respiratory problems in young pigs [7,8]. PRRSV infection can induce several immunosuppressive responses [9], such as: i) dysregulation of NK cell cytotoxic activity [10]; ii) poor production of IFN-alpha [11]; and iii) promotion of the secretion of immunosuppressive cytokines such as interleukin-10 (IL-10) and transforming growth factor-beta [10,12,13].

Systematic vaccination and non-vaccination stamping-out are the two main strategies to control CSF [6,14]. Due to the enormous costs of stamping-out, systematic vaccination is a more effective strategy for CSF control in CSF endemic areas [6]. Two major kinds of CSFV vaccines, the modified live vaccine (MLV) and the subunit vaccine, are widely used in many countries [6,14]. The MLV vaccine can induce not only humoral immune responses but also cell-mediated immune responses against virulent CSFV. Subunit vaccines, such as E2 vaccines, usually only induce antibody responses [14]. However, the disadvantages of CSFV MLV vaccines are that their efficacy is inhibited by maternal-derived antibody (MDA) [14,15,16,17,18,19] and they lack differentiation with infection from vaccinated animals (DIVA) according to serological assays [16,20]. The CSFV subunit vaccines based on the E2 protein allow DIVA by E^rns^ enzyme-linked immunosorbent assays and provide good protection [21,22,23]. The drawbacks of E2 subunit marker vaccines are that they induce protection later than MLV vaccines, and their efficacy also interferes with maternal antibodies [21,24].

Transplacental transmission of CSFV occurred before the onset of the antibody response when sows were challenged with either high- or low-virulence CSFV strains. Therefore, rapid and solid immunity after sow vaccination is required for the prevention of congenital viral persistence [3]. In Taiwan, CSFV MLV has been used since the 1950s and proven to be sufficiently protective. CSFV MLV vaccination is only recommended in sows at 4 weeks post-farrowing (nonpregnancy stage) to overcome persistent infection. To avoid MDA, which interferes with the efficacy of CSFV MLV in the clinic, piglets should be vaccinated at 6 and 9 weeks old when sows are vaccinated in the nonpregnancy stage. However, PRRSV is still a major problem and difficult to control in the nursery stage, which overlaps with the CSFV MLV vaccination period in Taiwan. Certain severe PRRS cases in the nursery were observed just after CSFV MLV vaccination (data from the Animal Diseases Diagnostic Center of National Pingtung University of Science and Technology, not shown). The most reasonable explanation for PRRS and porcine respiratory disease complex induction is stress, which would be caused not only by vaccination but also by the side effects of CSFV MLV, pathogens spread by needles, the synergistic effects of bacterial pathogens such as *Glaesserella parasuis* (*G. parasuis*, previously called *Hemophilus parasuis*) and other factors [25]. In contrast, the E2 vaccine is recommended for application in sows at 4–5 weeks pre-farrowing and elicits a high level of neutralizing antibody [26,27,28], whereas the vaccination of offspring can be delayed until they are 10–12 weeks old [28], which is when most piglets have recovered from PRRS. Therefore, the level of MDA is very important for CSFV vaccination programs in piglets. Previous research findings showed that PRRSV infection prior to CSFV vaccination significantly suppressed the antibody response [29,30]. In addition, CSFV immunization during the acute phase of PRRSV infection could result in vaccination failure [31]. However, the correlation between the CSFV MDA levels produced in response to different types of CSFV vaccines and the PRRSV load in the field remains to be investigated. Herein, this retrospective study aimed to elucidate the sero-dynamics of the CSFV and PRRSV loads in piglets born from sows immunized with different types of CSFV vaccines to further the understanding of the interactions between the CSFV vaccine and the PRRSV, which is still prevalent in most areas of intense pork production in the field.

## 2. Results

### 2.1. Levels of CSFV Antibody in Pigs of Different Ages from Sows Immunized with Different Types of CSFV Vaccines

**Figure 1:**
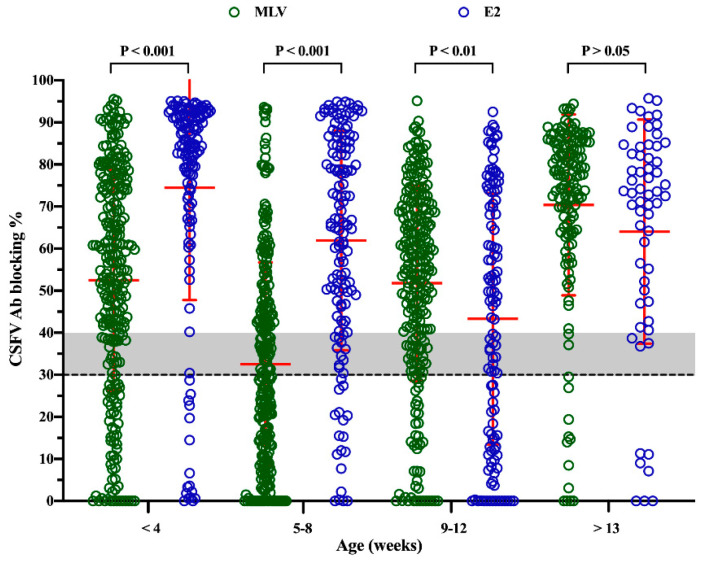
The humoral responses based on ELISA in piglets born in the classical swine fever virus modified live vaccine, (CSFV MLV) and E2 vaccine groups. The results are expressed as blocking %. The dashed line indicates a blocking %, the threshold below which the samples were considered negative. A blocking % between 30 and 40 was interpreted as suspected (gray area). A blocking % greater than or equal to 40 was interpreted as positive. The error bars show the standard deviation (SD). *P* values < 0.05, < 0.01 and < 0.001 were considered statistically significant, highly significant and very highly significant, respectively.

A total of 1398 blood samples from 42 PRRSV-positive commercial herds were included in this study that were obtained from 943 cases (from 29 pig herds) from the MLV group together with 455 cases (from 13 pig herds) from the E2 group. The evaluation of CSFV antibody levels at different ages in pigs revealed that the level of CSFV antibody was very highly significant (*P* < 0.001), higher in the E2 group than in the MLV group when pigs were less than 4 weeks old and 5–8 weeks old (Figure 1). However, the level of CSFV antibody in the MLV group was significantly higher than that in the E2 group at 9–12 weeks of age (*P* < 0.01), but the difference between the two groups was not statistically significant after 13 weeks of age (Figure 1).

### 2.2. Correlation of the PRRSV Load and Level of CSFV MDA in the Piglets without CSFV Vaccination from Different Groups

**Figure 2:**
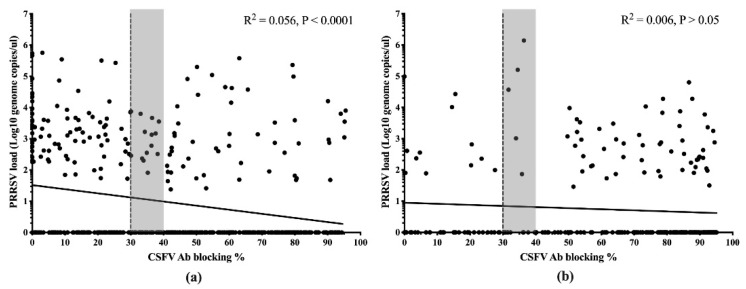
Linear regression analysis was used to calculate the porcine reproductive and respiratory virus (PRRSV) loads and levels of CSFV maternal-derived antibody (MDA) in piglets without CSFV vaccination in the MLV (**a**) and E2 (**b**) groups. The dashed line indicates a blocking %, the threshold below which the samples were considered negative. A blocking % between 30 and 40 was interpreted as suspected (gray area). A blocking % greater than or equal to 40 was interpreted as positive. *P* values < 0.05, < 0.01 and < 0.001 were considered statistically significant, highly significant and very highly significant, respectively.

To examine the correlation of the PRRSV load and level of CSFV MDA in different groups, the PRRSV load of all serum samples was quantitated by real-time polymerase chain reaction (PCR). The presence of PRRSV was calculated only in the piglets without CSFV vaccination. A total of 802 samples fit this criterion. The correlation between the PRRSV load and the level of CSFV antibody in piglets without CSFV vaccination was calculated using a linear regression analysis. The results of MLV group showed that there was a very highly significant negative correlation between the PRRSV load and the level of CSFV antibody (*P* < 0.0001) (Figure 2a). Surprisingly, there was no significant correlation between the PRRSV load and the level of CSFV antibody in piglets from E2 group (*P* > 0.05) (Figure 2b).

### 2.3. Viral Load and Detection Rate of PRRSV in the Piglets without CSFV Vaccination from Different Groups

**Figure 3:**
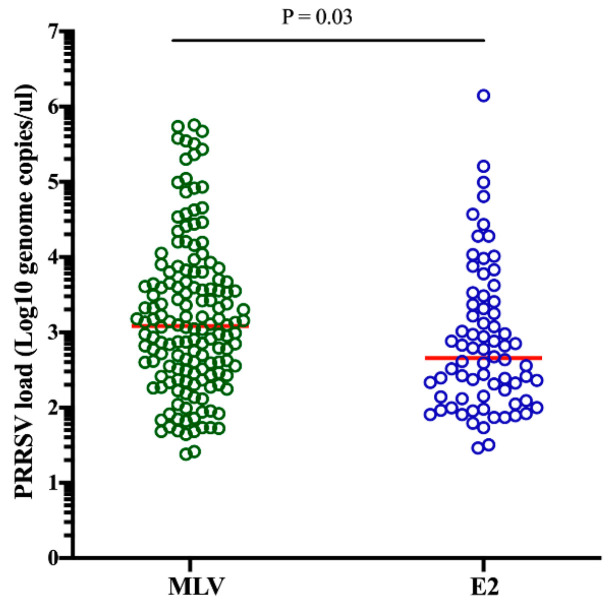
PRRSV loads in the serum samples from both the CSFV MLV and E2 vaccinated groups. The red horizontal lines represent the median concentrations for each group. Unpaired, 2-tailed Student’s *t*-tests were used to compare the PRRSV loads between the MLV and E2 groups. *P* values < 0.05, < 0.01 and < 0.001 were considered statistically significant, highly significant and very highly significant, respectively.

In the piglets without CSFV vaccination in different groups, 152 of 517 samples (29.40%) from the MLV group and 72 of the 285 samples (25.26%) from the E2 group were positive for PRRSV. The difference in the detection rate of PRRSV in the piglets without CSFV vaccination between both groups was not statistically significant (*P* = 0.24). However, the PRRSV load was significantly higher (ranging from 1.38 to 5.75 log10 PRRSV genome/µL, median 3.09 log10) in the MLV group than in the E2 group (ranging from 1.46 to 6.15 log10 PRRSV genome/µL, median 2.66 log10) (*P* = 0.03) (Figure 3).

### 2.4. Viral Load and Detection Rate of PRRSV in Different Age Groups

**Figure 4:**
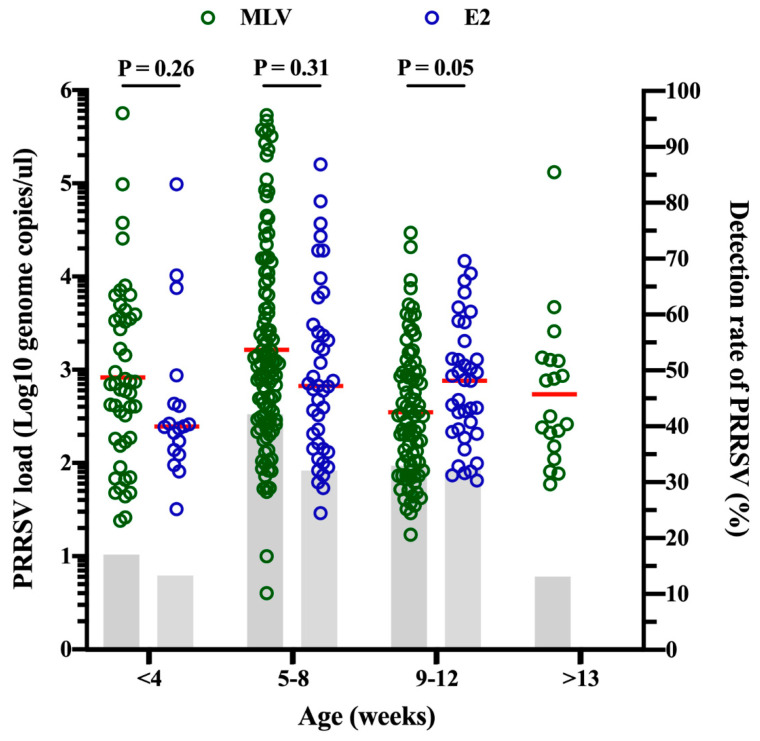
PRRSV loads (dot, left Y axis) and detection rates of PRRSV (bar chart, right Y axis) in serum samples from pigs of different ages from both the CSFV MLV and E2 groups. The dots represent the individual PRRSV load of each pig. The red horizontal lines represent the median concentrations for each group. The bar represents the detection rate of PRRS in pigs of different ages. An unpaired, 2-tailed Student’s *t*-test was used to compare the PRRSV loads between the MLV and E2 groups. *P* values < 0.05, < 0.01 and < 0.001 were considered statistically significant, highly significant and very highly significant, respectively.

The evaluation of the PRRSV load at different ages revealed that the mean PRRSV load was not significantly different for both vaccine types in piglets that were less than 4 weeks old, 5–8 weeks old and 9–12 weeks old (Figure 4). Surprisingly, no PRRSV viremia was found in pigs aged more than 13 weeks in the E2 group (Figure 4 and Table 1). Furthermore, we compared the detection rate of PRRSV at different ages for different types of CSFV vaccines. The details of the detection rate of PRRSV for different types of CSFV vaccines are shown in Table 1. The highest detection rate of PRRSV in pigs was found at 5–8 weeks old (37.81%) compared to that found at other ages (Table 1). At 5–8 weeks old, the detection rate of PRRSV was significantly higher in the pigs from the MLV group (42.15%) compared with that in pigs from the E2 group (29.79%) (*P* = 0.019). The overall detection rate of PRRSV was significantly higher in the pigs from the MLV group than in that in pigs from the E2 group (*P* = 0.011) (Table 1).

## 3. Discussion

In industrial pork production, multiple viral infections, either in an individual pig or in a herd, with or without bacterial complications have occurred regularly. The systemic application of CSFV MLV further complicates the situation. In order to avoid this background noise, a total of 1398 cases were collected from commercial pig herds for statistical analysis. This study explored the intersectional plane of the interaction between PRRSV and CSFV to shed light on the day-to-day situation in the field.

In areas without CSFV eradication, such as Taiwan, routine vaccination is one of the most effective strategies for the prevention and control of this disease. Two major CSFV vaccines, the MLV and E2 vaccines, are recommended [6,14]. The CSFV MLV vaccine can induce not only humoral immune responses but also cell-mediated immune responses against virulent CSFV [14]. However, several disadvantages of CSFV MLV vaccines have been identified: i) a lack of DIVA according to serological assays [16,20]; ii) the adverse effects of the CSFV MLV vaccine in vaccinated pigs [32,33]; iii) pig-to-pig transmission of MLV [32]; and iv) farm-to-farm transmission of MLV by vehicles [32]. Additionally, the influence of MDA on the efficacy of CSFV MLV in the field has been well discussed [14,15,16,17,18,19]. Therefore, there is a negative correlation between the levels of MDA and CSFV MLV efficacy [16,17,19]. In Taiwan, CSFV MLV vaccination is only recommended in sows at 4 weeks post-farrowing to overcome persistent infection. Our results showed that the lowest level of CSFV antibody in piglets was found at 5–8 weeks of age in the MLV group (Figure 1), which suggests a time window that may be appropriate for CSFV vaccination. In addition to MDA, several other immunosuppressive viruses, such as PRRSV [29,30,31], PCV2 [34,35,36] and pseudorabies virus [37], can potentially interfere with the efficacy of the CSFV MLV vaccine.

The immunosuppression caused by PRRSV is related to IL-10 stimulation and inflammatory cytokine downregulation [10,12,13,30]. It has also been shown that vaccine failure can occur when CSFV MLV vaccine strain replication is inhibited by tumor necrosis factor-alpha induced by PRRSV [30]. Previous studies demonstrated that CSFV MLV immunization during the acute phase of PRRSV infection could suppress the efficacy of CSFV vaccination [29,30,31]. Thus, the CSFV vaccination time should not overlap with the PRRSV infection period. The efficacy of the CSFV MLV vaccine in the field is worthy of further investigation for the purpose of CSF eradication. According to the diagnostic reports of the Animal Diseases Diagnostic Center of National Pingtung University of Science and Technology, some severe PRRS cases in nursery pigs occurred just after CSFV MLV vaccination (data not shown). In industrial pork production, pigs often have multiple viral infections (e.g., PRRSV + PCV2) together with complicated bacterial infections such as *G. parasuis* [38]. A study revealed that infection with multiple viruses, such as PRRS and PCV2, may affect the replication or viral activity of the CSFV MLV virus [39]. PCVAD cause multifactorial syndromes that have been be a major problem in Taiwan [40,41]. Fortunately, the problems caused by PCV2 have resolved significantly since the PCV2 vaccine became available in Taiwan [42]. Currently, PRRSV remains a major problem and is difficult to control in the nursery stage, as indicated by the overall detection rate of PRRSV in pigs being the highest at 5–8 weeks of age (37.81%) compared to that at other ages (Table 1). Taken together, it should be further considered that the efficacy of CSFV MLV and stress caused by CSFV MLV vaccination during the PRRSV susceptibility period may induce clinical signs of PRRSV infection.

In the E2 group, the lowest level of CSFV antibody was observed at 9–12 weeks of age (Figure 1). To enhance CSFV vaccine efficacy, this time window may be more appropriate for CSFV vaccination in piglets. Based on the results of E2 vaccination in sows at 4–5 weeks pre-farrowing, the vaccination time of the offspring can be delayed until they are 10–12 weeks old [28], which is when they are less susceptible to PRRSV, as observed in most Taiwanese pig herds. In addition, the level of CSFV antibody was significantly higher at the suckling stage (less than 4 weeks old) in the E2 group than in the MLV group (Figure 1), reflecting the different levels of CSFV antibody in sows. This difference was due to sows being vaccinated with E2 at 4–5 weeks pre-farrowing, which elicited a sufficiently high level of CSFV antibody (Figure 1) [27,28]. However, decreases in CSFV antibody titers should be considered when sows are vaccinated with the MLV vaccine post-farrowing and/or when some sows have returned several times. Although the E2 vaccine can only induce antibody responses without inducing cellular immunity, the shedding of vaccine antigens in the field should not be a concern. In addition, the E2 vaccine induces E2-specific neutralizing antibodies to protect different genotypes from highly virulent CSFV challenge [23]. Although there may be other background issues (such as hygiene status and management of the herds) which co-influence the piglets’ health state according to previous studies, CSFV E2 subunit vaccine on sows has shown several benefits, such as: (i) the detection rate of CSFV nucleic acid in saliva in their offspring was dramatically decreased [43]; (ii) the efficient induction of high levels of CSFV antibody until slaughter when their offspring only received a single shot of CSFV immunization [26]; and (iii) increase in the survival rate of the nursery pigs in our analyzed herds (herd practitioners’ observation), which are consistent with our interpretation of the results in this study. Finally, and most importantly, the major difference between CSFV MLV vaccine and E2 subunit vaccine or inactivated vaccine is that only CSFV MLV provides replicating antigen [14].

In conclusion, our study revealed the sero-dynamics of piglets born from sows vaccinated with the CSFV MLV and E2 vaccines. There was a very highly significant negative correlation between the PRRSV load and the level of CSFV antibody in the CSFV MLV group. The lowest level of CSFV antibody was observed in the CSFV MLV group at 5–8 weeks of age, during which pigs are highly susceptible to PRRSV and CSFV MLV vaccination should be avoided. In contrast, after E2 vaccination of sows at 4–5 weeks pre-farrowing, the level of CSFV antibody remained positive at 9–12 weeks, which allowed CSFV vaccination with MLV to be postponed to avoid an overlap with the PRRS susceptibility period at 5–8 weeks of age. Additionally, our findings indicate that the vaccination of CSFV MLV in piglets during the PRRSV susceptibility period at 5–8 weeks of age may be overloading the piglet’s immune system and should be a critical concern for industrial pork production in the field. Thus, using vaccines that provide non-replicating antigen such as E2 subunit vaccines is recommended.

## 4. Materials and Methods

### 4.1. Sample Source and Processing

Blood samples were collected in BD Vacutainer tubes with clot activator and gel (BD Diagnostics, Plymouth, UK) from piglets and submitted to the Animal Diseases Diagnostic Center of National Pingtung University of Science and Technology, Taiwan. All piglets were divided into two groups, the MLV and E2 groups. Piglets in the MLV group were born from sows vaccinated with the CSFV MLV vaccine at 4 weeks post-farrowing, whereas piglets in the E2 group were born from sows vaccinated with the CSFV E2 subunit vaccine (Bayovac^®^ CSF-E2, Bayer Animal Health) at 4–5 weeks pre-farrowing. The blood samples were centrifuged at 2150× *g* for 15 min with a Himac CF 9RX (Hitachi Koki, Tokyo, Japan), and then the sera were carefully transferred into 1.5 mL centrifuge tubes. The stock serum was kept at −80 °C until needed.

### 4.2. Sample Preparation and PRRSV Real-Time PCR

Nucleic acid extraction was performed with a MagNA Pure LC 2.0 instrument by using the MagNA Pure LC total nucleic acid isolation kit (Roche Applied Science, IN, USA). cDNA synthesis was performed using PrimeScriptTM RT reagent kits (Takara, Kyoto, Japan). To quantify the PRRSV load in serum samples, a ZNA probe-based real-time PCR assay was tested and performed with a LightCycler^®^ 96 System (Roche Applied Science, Basel, Switzerland) [44].

### 4.3. Serologic Assessment

The serum concentration of CSFV antibody was evaluated by a commercial ELISA (HerdChek CSFV antibody ELISA test kit, IDEXX, ME, USA). After measuring the optical density at a wavelength of 450 nm (OD450) with a Biochrom Anthos Zenyth 200st spectrophotometer (Anthos Labtec Instruments, Salzburg, Austria), if the mean OD450 of the duplicate negative controls (NC$\bar{x}$) was more than 0.5 and the mean blocking % of the duplicate positive controls was greater than 50, the assay was considered valid. The blocking % was calculated with the equation, $\mathrm{Blocking}\;\%=100\times \frac{\mathrm{NC}\bar{x}-\mathrm{Sample}\;\mathrm{OD}450}{\mathrm{NC}\bar{x}}$. A blocking % less than or equal to 30 was interpreted as negative. A blocking % between 30 and 40 was interpreted as suspected. A blocking % greater than or equal to 40 was interpreted as positive.

### 4.4. Statistical Analysis

Student’s *t*-test was applied to assess differences in the PRRSV load in the different conditions between the two groups. The relationship between the PRRSV load and the blocking percentage of the CSFV antibody was analyzed by linear regression. Positive rates of PRRSV in different age groups were determined with the chi-square test with Yate’s correction. *P* values < 0.05, < 0.01 and < 0.001 were considered statistically significant, highly significant and very highly significant, respectively.

## Figures and Tables

**Table 1:** Detection rates of PRRSV in the serum samples from pigs at different ages from both the CSFV MLV and E2 groups.

Age (Weeks)	CSFV Vaccine Type		Total	*P* Value ^†^
	MLV	E2		
<4	47/276 (17.03)	18/135 (13.33)	65/411 (15.82)	0.412
5–8	110/261 (42.15)	42/141 (29.79)	152/402 (37.81)	0.019
9–12	86/261 (32.95)	37/119 (31.09)	123/380 (32.37)	0.081
>13	19/145 (13.10)	0/60 (0)	19/205 (9.27)	N/A *
Total	262/943 (27.78)	97/455 (21.32)	359/1398 (25.68)	0.011

^†^*P* values < 0.05, < 0.01 and < 0.001 were considered statistically significant, highly significant and very highly significant, respectively. * N/A, not applicable; the chi-square calculation does not support cell values that are zero.
